# Novel Glial Cell Functions: Extensive Potency, Stem Cell-Like Properties, and Participation in Regeneration and Transdifferentiation

**DOI:** 10.3389/fcell.2020.00809

**Published:** 2020-08-18

**Authors:** Valentin Milichko, Vyacheslav Dyachuk

**Affiliations:** ^1^Department of Nanophotonics and Metamaterials, ITMO University, St. Petersburg, Russia; ^2^National Scientific Center of Marine Biology, Far Eastern Branch, Russian Academy of Sciences, Vladivostok, Russia

**Keywords:** Schwann cell precursors, peripheral glial cells, heterogeneity, plasticity and multipotency, reprogramming, neurological diseases, carcinogenesis

## Abstract

Glial cells are the most abundant cells in both the peripheral and central nervous systems. During the past decade, a subpopulation of immature peripheral glial cells, namely, embryonic Schwann cell-precursors, have been found to perform important functions related to development. These cells have properties resembling those of the neural crest and, depending on their location in the body, can transform into several different cell types in peripheral tissues, including autonomic neurons. This review describes the multipotent properties of Schwann cell-precursors and their importance, together with innervation, during early development. The heterogeneity of Schwann cells, as revealed using single-cell transcriptomics, raises a question on whether some glial cells in the adult peripheral nervous system retain their stem cell-like properties. We also discuss how a deeper insight into the biology of both embryonic and adult Schwann cells might lead to an effective treatment of the damage of both neural and non-neural tissues, including the damage caused by neurodegenerative diseases. Furthermore, understanding the potential involvement of Schwann cells in the regulation of tumor development may reveal novel targets for cancer treatment.

## Introduction

Peripheral glial cells encompass a wide variety of cell types, including myelinating Schwann cells, non-myelinating Schwann (Remak) cells (Jessen and Mirsky, 2010), glia of the enteric nervous system (Ochoa-Cortes et al., 2016), satellite glial cells of peripheral ganglia, and perisynaptic Schwann cells (PSCs) located in neuromuscular junctions (Alvarez-Suarez et al., 2020). In addition, boundary cap cells give rise to terminal glia associated with dermal nerve endings (Zujovic et al., 2011; Gresset et al., 2015). Moreover, researchers have described other small subpopulations of glial cells, such as olfactory ensheathing cells that envelop bundles of axons in the olfactory bulb (Su and He, 2010) and specialized subtypes of glial cells in sensory organs (Ruffini endings, Krause end bulbs, and Meissner’s and Pacinian corpuscles) (Byers, 1985; Maeda et al., 1999; Kastriti and Adameyko, 2017).

Classically, peripheral glial cells have been viewed as multifunctional, nourishing, supporting and protecting neurons, myelinating axons, regulating synaptic connectivity and sensory function, and participating in the maintenance and regeneration of the peripheral nervous system (Barres, 2008). In recent decades, researchers have discovered that glial cells also play additional novel and unexpected roles, both during development and in adult animals (Adameyko et al., 2009; Dyachuk et al., 2014; Kaukua et al., 2014; Abdo et al., 2019; Xie et al., 2019), including participation in diseases (diabetes, tunnel syndrome, nerve paralysis, Guillain-Barre syndrome) (Gonçalves et al., 2018).

### Involvement of Schwann Cell Populations in Development

**FIGURE 1:**
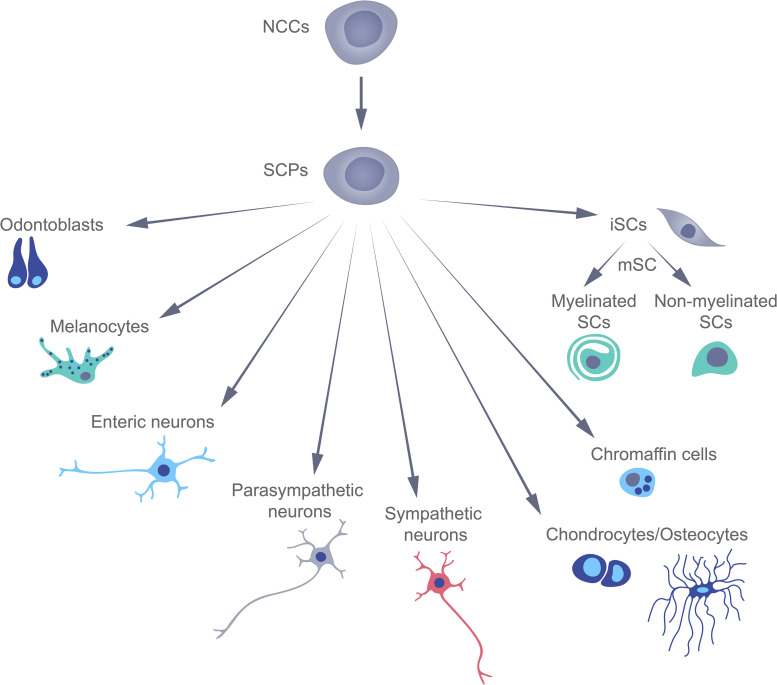
The multipotency of Schwann cell precursors during early mammalian development.

Traditionally, terminally differentiated peripheral glial cells (or Schwann) cells (SCs) have been thought to derive directly from multipotent neural crest cells (NCCs), which appear for only a short time during early mammalian development, and then give rise to a variety of other cell types (Le Douarin and Kalcheim, 1999). However, this concept has been challenged by several recent studies involving lineage tracing that have revealed the existence of Schwann cell precursors (SCPs) with transcriptional profiles similar to those of SCs and NCCs (Adameyko et al., 2009; Kastriti and Adameyko, 2017). These SCPs can differentiate into an extraordinary variety of cell types, including immature Schwann cells, which subsequently differentiate into the myelinating and non-myelinating Schwann cells, associated with peripheral nerves. Recent data, showed that embryonic Schwann cells are able to differentiate into endoneurial fibroblasts (Joseph, 2004), pigment cells (Adameyko et al., 2009), parasympathetic neurons (Dyachuk et al., 2014), mesenchymal dental cells (Kaukua et al., 2014), enteric neurons (Espinosa-Medina et al., 2017), chromaffin cells of the adrenal gland (Furlan et al., 2017), cells of the Zuckerkandl organ (Kastriti et al., 2019), chondrocytes, and osteocytes (Xie et al., 2019; Figure 1).

Are Schwann cell precursors needed, in association with early development, when a population of NCCs, that appears earlier, can produce a wide range of cell types? One fundamental difference between these two types of cells is that NCCs migrate along a concentration gradient of growth factors released by neighboring tissues during a specific 24 h period, whereas SCPs are continuously associated with rapidly growing embryonic nerves, migrating together with them to quickly reach, even distant, regions of the body. The relatively large size of mouse and chicken embryos requires a rapid and appropriately timed delivery of undifferentiated cells to various locations where they give rise to several specialized types of cells, a function that SCPs are capable of performing.

In this context, the mechanism(s) by which SCPs attach and detach from growing nerves and the temporal and spatial regulation of these mechanism(s) remain(s) unclear. It is known that the signals released by axons, as well as the actual physical interaction of axons with SCPs, are involved in the survival and differentiation of SCPs (Birchmeier and Nave, 2008), influencing their subsequent differentiation into specialized cell types (Dyachuk et al., 2014). The signaling pathway involving the neuregulin–ErbB heteromeric receptor plays crucial roles in both SCP migration and in the survival of glial cells (Birchmeier, 2009). For example, in teleost fish, the association of SCPs with nerves and their glial commitment are controlled by this pathway (Garratt et al., 2000; Honjo et al., 2008). Moreover, similar mechanisms are active in the mammalian peripheral nervous system (Britsch et al., 1998; Jessen and Mirsky, 2005; Dyachuk et al., 2014), as demonstrated by the observation that, in mice in which *ErbB2* has been genetically ablated, no SCPs are associated with the developing nerves, ultimately resulting in the widespread death of both motor and sensory neurons. *Sox10, ErbB3*, or *Nrg1* genes are responsible for the survival of SCPs, and the inactivation of these genes led to the degeneration of motor and sensory neurons (Riethmacher et al., 1997; Wolpowitz et al., 2000; Britsch et al., 2001). Genetic ablation of peripheral nerves in mouse embryos or the pharmacological impairment in zebrafish larvae depleted SCPs nerve-associated SCPs, thereby preventing the appearance of neurons of the PNS and of melanophore stem cells (Dooley et al., 2013).

Comparative single-cell transcriptomic analysis of NCCs and SCPs has revealed that these two embryonic cell populations express many common transcription factors (TF) (Kastriti and Adameyko, 2017; Soldatov et al., 2019). As shown before, during early differentiation, SCPs programming is downregulated, while neuronal, neuroendocrine (e.g., chromaffin cells), or mesenchymal (odontoblasts, chondrocytes, and osteocytes) traits are upregulated (Dyachuk et al., 2014; Kaukua et al., 2014; Furlan et al., 2017; Xie et al., 2019). What determines the specialization direction in which a SCP will develop remains unclear. Are the different nerve and body locations of SCPs involved in their type of specialization? Perhaps the specific signals released by cells in the innervated target organs help to determine the fate of SCPs. Appropriately designed experiments are required to answer these fascinating questions.

### Natural (Adaptive) Reprogramming of Schwann Cells

**FIGURE 2:**
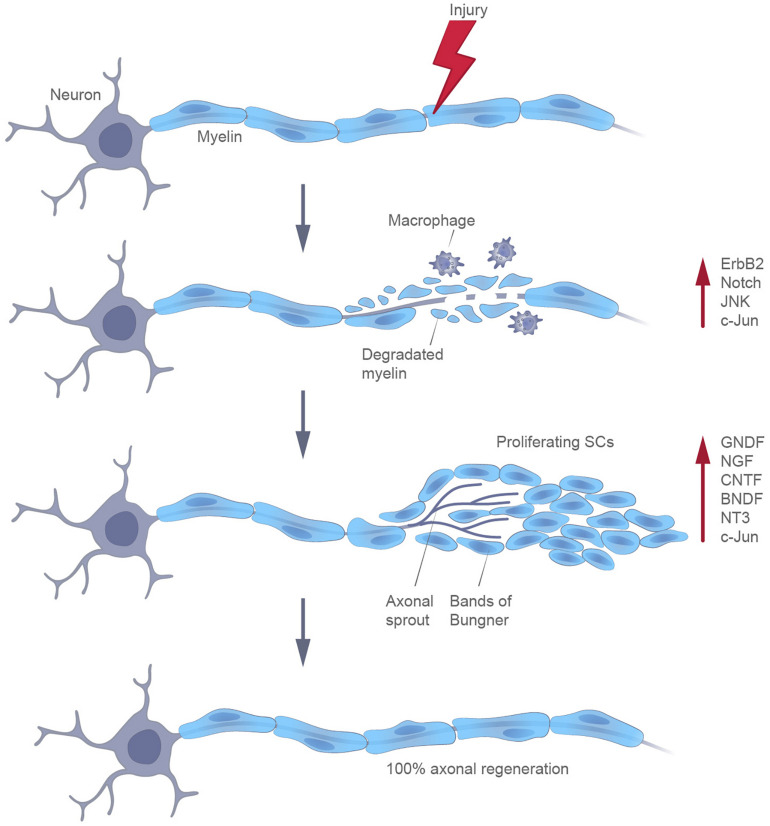
Participation of Schwann cells in the regeneration of peripheral axons, following injury.

Differentiated definitive somatic cells can be reprogrammed by enhancing the levels of the Yamanaka factors (Takahashi and Yamanaka, 2006). At the same time, specialized cells in certain adult mammalian tissues can be naturally reprogrammed in response to an injury (Merrell and Stanger, 2016). The most well-known example of such an adaptive reprogramming is the transformation of myelin cells into cells with a non-myelinating Schwann cell phenotype, following certain types of injuries of the nervous system. Schwann cells have a unique capacity to promote the recovery of axons. After detaching from their axons, these cells release neurotrophic factors that improve the axonal survival. Moreover, by radically changing the local signaling environment, they participate in the autophagy of myelin and in the expression of cytokines, being also able to attract macrophages for myelin clearance. Finally, SCs proliferate to replace the lost cells and differentiate to elongate, branch, and form regeneration tracks (Bungner bands) (Jessen et al., 2015; Figure 2). The molecular profiling of glia cells following injury is now receiving considerable attention, in order to determine their status.

Transcriptional profiling indicates that, following injury, Schwann cells acquire some properties of immature SCs, with concomitant repression of genes encoding proteins involved in the production of myelin (BrosiusLutz and Barres, 2014; Jessen and Mirsky, 2016). It should be emphasized that this transformation of mature Schwann cells into reparative Schwann cells is not actually dedifferentiation, although this process has been designated as such, previously. Indeed, this process involves the *de novo* expression of genes (*Shh* and *Olig1*) that are never expressed by embryonic Schwann cells, and whose expression is a unique aspect of the response to injury (Arthur-Farraj et al., 2012; Lin et al., 2015). At the same time, the transcription factor SOX2, a marker of developing SCPs, becomes upregulated as part of this reprogramming (Johnston et al., 2016).

The key molecular molecules that participate in this reprogramming of SCs include both certain transcription factors (e.g., c-JUN, SOX2) and signaling pathways (ERK, NOTCH). Moreover, the molecular characterization of bridge and distal stumps of transected nerves indicates that, during reprogramming, SCs acquire mesenchymal traits, i.e., this process is somewhat similar to an epithelial-to-mesenchymal transition (EMT) (Arthur-Farraj et al., 2017; Clements et al., 2017).

Epigenomic changes in Schwann cells play an important role in differentiation and in their myelination (Woodhoo et al., 2009; Arthur-Farraj et al., 2012; Napoli et al., 2012; Jessen et al., 2015; Ma et al., 2016). However, epigenomic reprogramming of SCs in response to injury has not yet been characterized in detail. For example, HDAC-mediated deacetylation of promyelinating transcription factor NF-κB is essential for the differentiation and myelination of SCs *in vitro* (Nickols et al., 2003; Chen et al., 2011). In contrast, the activation of NF-κB is not required for myelination of SCs *in vivo* (Morton et al., 2013). This discrepancy may indicate that the myelination of SCs during development, and following injury, is regulated by different transcriptional programs. Although NF-κB appears to regulate EMT genes, as shown for several human cancers (Pires et al., 2017), the underlying mechanism of the action NF-κB in glia development and myelination remains unknown.

Moreover, the findings concerning the levels of expression of TFs by SCs following injury are also in disagreement, sometimes. For example, some researchers have observed no changes in the levels of SC markers (Jessen et al., 2015), while others have found a decreased expression of transcription factors (Clements et al., 2017). Such discrepancies raise questions concerning the true nature of SCs during reprogramming. On the one hand, an upregulation through a transcriptional program orchestrated by c-JUN re-programs cells to become “repair” glial cells, with their own specific molecular signature (Arthur-Farraj et al., 2012); while on the other hand, the similarity of expression of transcription factors that is characteristic of EMT and of reprogrammed SCs after injury indicates their relationship to neural crest stem cells and/or to their own progenitors, SCPs or immature SCs.

**FIGURE 3:**
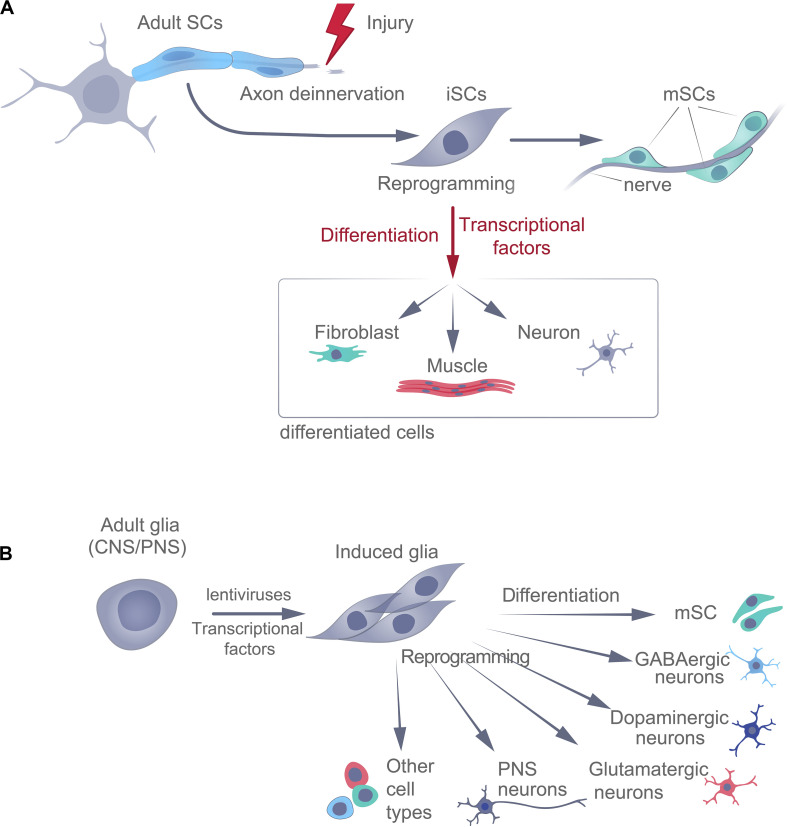
Naturally occurring (adaptive) **(A)** and experimental **(B)** reprogramming of adult glial cells, following injury. SCs, Schwann cells; iSC, immature Schwann cells; mSC, mature Schwann cells.

Of course, there is also the possibility that there are subpopulations of SCs that have been reprogrammed in different ways (mesenchymal, immature glial, and neural crest) at different sites of injury (Figure 3A).

Another function performed by SCs in adults is the participation in the regeneration of the tips of the digits. After injury of the ends of fingers or toes, the transcription factor SOX2 is upregulated in the SCs associated with the damaged nerves. These cells, then, detach from their axons and migrate into the regenerating region, where they secrete paracrine growth factors that induce the proliferation of the resident mesenchymal precursors (Johnston et al., 2016). Accordingly, genetic ablation of *Sox2* in SCs and denervation impairs the regeneration of nails and bones in mice and this regeneration can be rescued by transplantation of neonatal SCs (expressing SOX2) cultured *in vitro* into the injured digits (Le et al., 2005; Taranova et al., 2006; Johnston et al., 2016).

The skin is often used as a model system for research on regeneration, containing many different types of cells, including stem and progenitor cells. The skin of mice and humans is permeated by dense axons of a small diameter that are often severely damaged, in association with injuries of various kinds. In general, the recovery of thin axons in the skin resembles the analogous processes that occur in other parts of the PNS.

In injured skin, SCs detach from the nerves and undergo reprogramming and, later, division, in order to restore lost cells (Johnston et al., 2013). SCs expressing the glial markers SOX2 and S100b actively migrate into the healing dermis to aid in its repair, probably by forcing mesenchymal cells to proliferate (Johnston et al., 2013). DTA-dependent ablation of SOX2^+^-glial cells reduces the proliferation of dermal and epidermal cells, thereby impairing wound healing (Johnston et al., 2016).

Interestingly, SOX2/S100b/P75^+^ glial cells, which may participate in dermal maintenance and wound-healing, attach to axons surrounding the hair follicles (Hunt et al., 2008; Biernaskie et al., 2009), but this still remains unclear. Peripheral glial cells activated in response to injury can promote wound healing in the skin of adult mice. Such repairing SCs detach from the injured nerves and move into the granulation tissue, where they are reprogrammed into invasive mesenchymal-like cells and drive peripheral nerve regeneration (Clements et al., 2017; Parfejevs et al., 2018).

These examples of the participation of Schwann cells, together with axons, in the regeneration of nerves and target tissues, make these cells attractive for regenerative therapies. However, a more in-depth molecular understanding of these processes is required before clinical application can be considered.

Moreover, in zebrafish, Müller glia in the retina can differentiate into neurons in response to lesions (Raymond et al., 2006) through a process dependent on TF ASCL1 (Ramachandran et al., 2010). Thus, in addition to helping restore small damaged axons, forcing the proliferation of mesenchymal cells, and participating in dermal maintenance and wound healing, as well as in the regeneration of nails and bones, glial cells may be involved in adult neurogenesis. Reprogrammed iSCs may even be sources of other types of cells in adult vertebrates, in response to damage (Figure 3A).

### Experimental Reprogramming of Schwann Cells

Terminally differentiated somatic cells can be induced to become pluripotent (dedifferentiation) or switch to a different specialized phenotype (transdifferentiation). Experimental differentiation of SCs into neurons in adults is complicated and time-consuming and has not yet been standardized. Transdifferentiation involves reprogramming the cell, which directly switches the cellular phenotype to that of another somatic cell type (Figure 3B), e.g., the conversion of glial cells into neurons. This can be achieved experimentally, both *in vitro* and *in vivo*, using many different approaches – either indirectly (via iPSCs using TF or via iNPCs using TF^+^ differentiation factors) or directly (using pro-neuronal TF^+^ differentiation and maturation factors or chemicals), each with its own advantages and disadvantages. For example, in cell culture, a chemical cocktail containing inhibitors of HDACs, GSK-3 kinases, and TGF-β-mediated pathways, in combination with hypoxia, can reprogram fibroblasts into neuroprogenitor cells (Cheng et al., 2014). In addition, fibroblasts can be reprogrammed directly into neurons using small molecules alone (Li et al., 2014; Hu et al., 2015). In connection with these promising procedures for the transformation of somatic cells, the cells obtained still need to be genetically and functionally characterized, and these experimental protocols require standardization.

To reprogram cells using regulatory factors, bacterial or viral vectors are introduced into cells to cause an overexpression of key transcription factors, and thereby, initiate transdifferentiation (Patel and Yang, 2010; Grath and Dai, 2019); CRISPR/Cas9 gene editing can also employed to alter the pattern of gene expression directly (Rubio et al., 2016); or drugs that target transcription factors can be used to give rise to epigenetic remodeling (e.g., for transdifferentiation of human fibroblasts into endothelial cells, cardiac cells into skeletal myocytes, and mesenchymal stem cells into cardiomyocytes) (Naeem et al., 2013; Kaur et al., 2014; Sayed et al., 2015). Of these three approaches, the direct reprogramming of neuronal cells by introduction of lentiviral vectors that results in an overexpression of specific transcription factors is the most popular and effective, at present. Examples of cells that have been transdifferentiated into neurons, to date, include glia (Heins et al., 2002), mouse and human fibroblasts (Vierbuchen et al., 2010; Yoo et al., 2011; Adler et al., 2012), and fibroblasts and astrocytes (Addis et al., 2011; Caiazzo et al., 2011; Rivetti Di Val Cervo et al., 2017).

The transcription factor PAX6 is a key determinant of the neurogenic potential of glial cells from the cortex (Heins et al., 2002). In the case of transformation of glia (astrocytes) from the CNS into neurons, both *in vitro* and *in vivo*, the most common approach involves TFs, such as NEUROG2, BRN4, NEUROD1, ASCL1, DLX2, and/or SOX2 (Addis et al., 2011; Guo et al., 2014; Liu et al., 2015; Brulet et al., 2017; Table 1).

**TABLE 1:** Transcription factors utilized for *in vivo* reprogramming of glial cells into neurons in the CNS.

Type of CNS cells reprogrammed	Transcription factors	Other factors added	Cells generated	References
Astrocytes	PAX6	None	Neurogenic cells	Heins et al., 2002
Astrocytes	NEURO2	FGF+EGF	Dopaminergic/glutaminergic neurons	Grande et al., 2013
Reactive astrocytes and NG2 glia	NEUROD1	None	Glutaminergic neurons	Guo et al., 2014
NG2 glia	SOX2	None	GABAergicneurons	Heinrich et al., 2010
Astrocytes	ASCL1	None	Glutamatergic and GABAergic neurons	Liu et al., 2015
NG2 glia	ASCL1 + LMX1a + NURR1	None	Glutamatergic and GABAergic neurons	Torper et al., 2013
Astrocytes	NEUROD1 + ASCL1 + LMX1a	miR218	Dopaminergic neurons	Rivetti Di Val Cervo et al., 2017
Astrocytes	NEUROD1	None	Neurons	Brulet et al., 2017
Astrocytes	SOX2	VPA	GABAergic neurons	Su et al., 2014
Astrocytes	SOX2	VPA + BDNF + NOGGIN	GABAergic, glycinergic, serotonergic, cholinergic neurons	Wang et al., 2016

In general, SCs that support the repair of damaged nerves are too short-lived to complete this process successfully. Consequently, considerable research is now focused on genetic reprogramming designed to allow SCs to retain their ability to repair damaged axons. Adenoviral vectors were the first to be successfully utilized to transduce Schwann cells in a peripheral nerve (Shy et al., 1995) and, later, lentiviral vectors were found to be even better for this purpose (Naldini et al., 1996).

Lentiviral-mediated overexpression of the trophic factor GDNF by both the intact and lesioned rat sciatic nerve elevates the density of the present SCs, but, at the same time, the morphology of these cells becomes abnormal and myelination of axons is severely impaired (Eggers et al., 2013). Moreover, although GDNF promotes the growth of local axons, the trapping of axons in the nerve (“candy store” effect) is detrimental to regeneration (Eggers et al., 2013; Santosa et al., 2013).

By employing a lentiviral vector to induce the overexpression of the neurotrophin NGF in SCs of the rat sciatic nerve, 2 weeks after injury, elevated the number of axons expressing NF200, ChAT, and CGRP and improved axonal regeneration (Shakhbazau et al., 2012). When SCs were induced to express BDNF, CNTF, or NT3, in an attempt to repair unilateral 1-cm defects in rat peroneal nerves, the differential effects on the morphology of peripheral nerve grafts, the number and type of regenerating axons, myelination, and locomotor function were observed, 10 weeks later (Godinho et al., 2013).

In general, glial cells are readily available and apparently suitable for direct reprogramming into neurons. Since the neurons and glia in the CNS originate from the same neural population, their epigenetic profiles are, presumably, the same. It is highly likely that experimenting with many more different types of adult cells and transcription factors will increase the yields of the target cells and, at the same time, lower the cost of future therapies.

### Glial Reprogramming *in vivo* in the Context of Neurological Diseases

At present, the progressive damage and death of neurons, which are post-mitotic and do not regenerate, is incurable. The source of the differentiated cells to be reprogrammed is important. Parenchymal astrocytes and NG2^+^ glia have been successfully reprogrammed into NEUN^+^ dopaminergic, glutaminergic, and GABA neurons of the striatum and cortex, using lentiviral vectors. These vectors have encoded a single TF (NEUROD, SOX2, or ASCL1) (Heinrich et al., 2010; Guo et al., 2014; Liu et al., 2015) or a mixture of these factors (ASCL1 + BRN2 + MYT1 or NEUROD + ASCL1 + LMX1a + miR218 or SOX2, together with the neurotrophic factors BDNF + NOGGIN, or NEUROG2 with FGF + EGF) (Grande et al., 2013; Torper et al., 2013; Rivetti Di Val Cervo et al., 2017; Table 1). Moreover, astrocytes from the spinal cord can be transdifferentiated into GABA-, glycin-, serotonin-, and cholinergic neurons through the overexpression of *Sox2*, together with the addition of valproic acid (which inhibits histone deacetylase), BDNF, and NOGGIN (Su et al., 2014; Wang et al., 2016; Table 1).

As indicated above, despite recent progress in increasing the yield of neurons from transformed glia, relatively few neurons have been obtained and these have survived only for a short period of time. These limitations may be overcome through the addition of other growth factors (e.g., FGF2, EGF) or/and chemicals (calcitriol, α-tocotrienol) (Grande et al., 2013; Gascón et al., 2016). In addition, appropriate alterations of the microenvironment (e.g., through co-expression of BDNF and NOG, or the presence of VPA) might also influence the maturation and survival of neurons *in vivo* (Niu et al., 2015; Wang et al., 2016).

In this context, reprogramming of glial cells, for example, in order to obtain new neurons during development, might also offer a viable approach to treatment *in vivo* (Bertrand et al., 2002; Dyachuk et al., 2014). One example of how this strategy has already been utilized successfully is in the transformation of spiral ganglion glial cells into auditory neurons to restore hearing (Noda et al., 2018). Direct conversion of glial cell into neurons of the CNS may eventually provide an effective treatment for various neurodegenerative diseases, such as ALS, Alzheimer’s (AD), and Parkinson’s (PD) disease. For example, in mice with PD, the treatment of astrocytes with a lentiviral vector that induced the overexpression of the transcription factors NEUROD1, ASCL1, LMX1A, and miR218, increased their numbers of dopaminergic neurons and led to the recovery of motor function, 5 weeks after treatment (Rivetti Di Val Cervo et al., 2017). In addition, in AD animal models, reactive glial cells can produce the transcription factor NEUROD1, which leads to the reprogramming of astrocytes into glutamatergic neurons, and of NG2 glial cells into glutamatergic and GABAergic neurons (Guo et al., 2014).

These examples of the reprogramming of glial cells into functional neurons are promising for the restoration of lost neurons, due to neurological damage or disease. In fact, *in vivo* reprogramming has several major advantages over *in vitro* approaches, including the maintenance of natural microenvironments, absence of complications associated with cell grafting, and a more efficient transdifferentiation. However, many questions remain unanswered. For example, whether transdifferentiation may be used to treat the cognitive and behavioral deficits that are becoming more common worldwide. Moreover, the problem of finding a more suitable source of cells with pronounced plasticity, rapid growth, and the ability to produce different types of neurons, still remains.

If reprogrammed cells are to be used successfully to treat neurodegenerative diseases, the neurons obtained must connect with other neurons to form functional pathways. In this context a significant achievement has been glial reprogramming into excitatory glutamatergic, inhibitory GABAergic, and dopaminergic neurons, which exhibit the electrophysiological properties of mature neurons, including action potential and synaptic connections (Grande et al., 2013; Torper et al., 2013; Guo et al., 2014; Su et al., 2014; Liu et al., 2015; Wang et al., 2016; Brulet et al., 2017; Rivetti Di Val Cervo et al., 2017).

In fact, although integration of “newborn” neurons into networks has been convincingly shown in several investigations, the full recovery of lost or damaged neuronal networks remains to be demonstrated. Clearly, much more insight into the mechanisms underlying neuronal transformation is required and single-cell deep sequencing, bioinformatics, and electrophysiological data should be of considerable value in this context.

### Reprogramming of SCs in the Tumor Milieu

**FIGURE 4:**
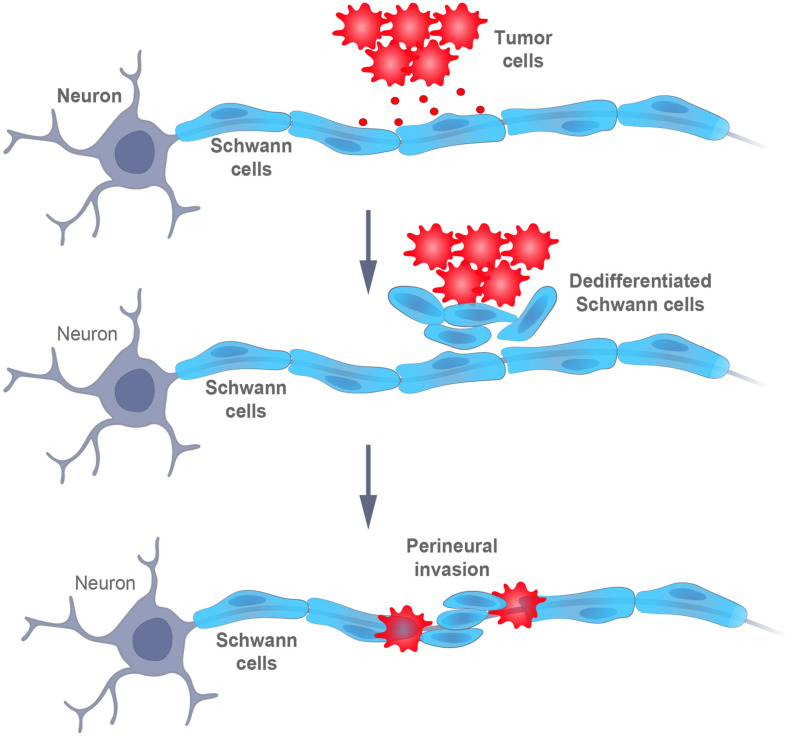
Schematic representation of the interaction between tumor and Schwann cells. Signals from the cancer cells promote the dedifferentiation of Schwann cells, which, then, intermingle with the cancer cells and promote their dispersal and, finally, perineural invasion (i.e., infiltration within or around the nerves).

For a long period, SCs were considered to not be involved in carcinogenesis and research on the promotion of cancer development, both *in vivo* and *in vitro*, has focused primarily on innervation by peripheral axons (Magnon et al., 2013). However, it is now clear that a dedifferentiated SCs phenotype, strongly reminiscent of those which arise in response to nerve injury, enables cancer progression (Deborde et al., 2016). In fact, SCs promote infiltration of peripheral nerves by cancer cells (perineural invasion, PNI) by establishing an early contact with these cells, thereby attracting them to the location of SCs (Demir et al., 2014; Figure 4).

More specifically, the motility and invasiveness of cancer cells depends on the secretion of chemokines by SCs and on the expression of the corresponding receptors on tumor cells (Deborde et al., 2016; Zhou et al., 2018). Moreover, SCs can degrade the extracellular matrix to form tunnels or bands coated with laminin, along which cancer cells migrate (Deborde and Wong, 2017). In addition, the release of BDNF by SCs promotes EMT in cancer cells, together with the loss of E-cadherin and the upregulation of NCAM1, thereby facilitating migration and invasion (Azam and Pecot, 2016; Deborde et al., 2016; Shan et al., 2016). Accordingly, SCs first attract cancer cells, and later, promote their invasion into nerves (Figure 4).

Interestingly, to promote their own perineural invasion, cancer cells exploit some of the canonical functions and features of SCs (plasticity, the ability to dedifferentiate in response to injury, alterations in the ECM), otherwise involved in neuronal repair and regulation. Genes expressed by pre-myelinating SCs, such as *Gfap, Ncam, L1-cam, P75, and Sox2*, have been implicated in this invasion (Demir et al., 2014, 2016). Thus, cancer cells can reprogram mature myelinated SCs into iSCs, which are more flexible and mobile (Jessen and Mirsky, 2019a). Moreover, as shown by Deborde et al. (2016), *Gfap* is expressed at high levels by dedifferentiated SCs, being closely associated with cancer cells, but not, at all, by differentiated Schwann cells, similarly, to the involvement of SCs in the repair of nerves (Jessen and Mirsky, 2019a).

At the same time, cancer cells interact with other types of normal cells as well, including immune cells that infiltrate the tumor and modulate its invasion (Shurin, 2012). Such recruitment of immune cells, which accelerates tumorigenesis, can be aided by SCs, in an analogous manner to the recruitment of immune cells during nerve repair (Deborde and Wong, 2017; Jessen and Mirsky, 2019b). Moreover, SCs modulate the activity of the immune cells that are, thus, recruited. For example, SCs exposed to melanoma cells *in vitro* enhance the ability of myeloid-derived suppressor cells (MDSCs) to suppress T cell proliferation (Martyn et al., 2019). In addition, the exposure of SCs to tumor cells elevates their expression of myelin-associated glycoprotein (MAG), which inhibits axonal growth and influences glia-axon interactions (Quarles, 2007). Indeed, MAG itself increases the immunosuppressive potential of MDSCs in a T cell inhibitory assay in a similar manner to that through which SCs are exposed to tumor cells (Martyn et al., 2019). Thus, factors released by cancer cells exert effects on SCs, which attract immune cells, that participate in the formation of the microenvironment which determines the course of tumor development.

## Conclusion

The findings summarized here indicate that SCs can be transformed into different types of cells, both ontogenetically under natural conditions and experimentally in the laboratory. The multipotent properties of early populations of SC precursors make them attractive candidates for correcting various developmental pathologies. Furthermore, the adaptive reprogramming of SCs, in connection with axonal regeneration, gives rise to glia that can be transformed experimentally into different cell types, opens up new approaches to regenerative therapy of traumatic brain and spinal cord injuries, as well as neurodegenerative diseases.

In addition, a more detailed understanding of the molecular mechanisms underlying the promotion of carcinogenesis by SCs, primarily by facilitating perineural invasion and dissemination, may allow the treatment designed to suppress the initial stages of cancer development. Such an approach could also provide an alternative to anti-cancer therapies that target nerve fibers, which are usually quite toxic. Finally, our evolving understanding of the role played by SCs in carcinogenesis may promote the development of efficient approaches to managing cancer pain syndrome.

## Author Contributions

VM and VD were responsible for the intellectual content of this work. VD wrote the manuscript. Both authors contributed to the article and approved the submitted version.

## Conflict of Interest

The authors declare that the research was conducted in the absence of any commercial or financial relationships that could be construed as a potential conflict of interest. The handling editor declared a past co-authorship with one of the authors VD.
